# Diagnostic accuracy of CBCT voxel values and calcaneal QUS in detecting low bone mineral density and their association with serum biomarkers

**DOI:** 10.5339/qmj.2026.31

**Published:** 2026-06-15

**Authors:** Neetha Harisha, Shailesh Mane, Amritha C, Rajeshwari G. Annigeri

**Affiliations:** 1 Department of Oral Medicine and Radiology, College of Dental Sciences, Davangere, Karnataka, India

**Keywords:** Bone mineral density, computed tomography cortical index, cone beam computed tomography, osteoporosis, quantitative ultrasound, vitamin D

## Abstract

**Background and objective:**

Osteoporosis poses significant risks to skeletal health and dental treatment outcomes. This study evaluated the diagnostic potential of cone beam computed tomography (CBCT) voxel values and radiomorphometric analysis in identifying low bone mineral density (BMD), exploring their correlation with calcaneal quantitative ultrasound (QUS) and serum biomarkers.

**Methods:**

A cross-sectional study was conducted on 86 participants aged over 40 years. QUS of the calcaneus was utilized to classify subjects into normal or osteopenic/osteoporosis groups. CBCT-derived voxel values (cortical and cancellous) and radiomorphometric indices were recorded alongside serum calcium and 25(OH) vitamin D levels. Statistical analyses included Pearson’s/Spearman’s correlations and t-tests to compare groups.

**Results:**

Participants with normal QUS T-scores exhibited significantly higher CBCT cortical (855.63 ± 71.38) and cancellous (439.90 ± 85.61) voxel values compared to osteoporotic individuals (*P* < 0.001). Strong positive correlations were found between QUS T-scores and CBCT voxel values (*r* = 0.766 for cortical; *r* = 0.789 for cancellous), as well as with serum vitamin D (*P* < 0.001). Serum calcium showed a weaker but significant correlation. CBCT-derived radiomorphometric indices did not significantly correlate with QUS T-scores. However, a strong negative correlation was observed between the qualitative computed tomography cortical index (CTCI) grading and QUS T-score (*P* < 0.001).

**Interpretation and Conclusion:**

CBCT voxel values and CTCI strongly correlate with QUS measurements and vitamin D levels, offering superior diagnostic utility compared to standard morphometric indices. These metrics may serve as effective adjunctive tools for opportunistic osteoporosis screening in dental settings, facilitating early diagnosis and risk management.

## 1. INTRODUCTION

Osteoporosis affects approximately 200 million people worldwide and around 50 million individuals in India. More than 4.5 million women above the age of 60 years suffer spinal fractures, and more than 2.5 million sustain hip fractures every year in India due to osteoporosis. Osteoporosis is considered a silent condition that entails a significant social and economic burden for a country like India.^1^ Normal calcium balance, together with normal vitamin D status, has been considered crucial for maintaining bone metabolism.^2,3^

The ability to measure bone strength using non-invasive and cost-efficient methods is important for evaluating fracture risk according to the osteoporosis severity, as well as for monitoring the early-stage stabilization of artificial implants following implantation in bone (e.g., dental or orthopedic implants).

Bone mineral density (BMD) at the proximal femur and lumbar spine by dual-energy X-ray absorptiometry (DEXA) has been considered the gold standard for the diagnosis of osteoporosis. DEXA scan’s precision depends on the operator, machine, and positioning requirements of the system,^2,3^ further, its high cost and high X-ray doses limit its use.^4,5^

Quantitative ultrasound (QUS) of the calcaneus is a potential alternative to DEXA that uses ultrasound waves to measure bone density. It is radiation-free and costs less than DEXA, while being portable and simple to operate. The association of DEXA and QUS tests has been reported to present a margin of confidence of 90% in specificity and sensitivity. So, calcaneus QUS can be considered as an effective alternative to DEXA.^6^

Cone beam computed tomography (CBCT) is a widely applied imaging method in dentistry for diagnosis and treatment planning purposes, which provides a three-dimensional representation of the maxillofacial skeleton with minimal distortion and improved image sharpness. Furthermore, the examination has a relatively low cost and low dose compared with computed tomography (CT) techniques.^7^

CBCT is routinely used in elderly patients for various oral conditions, and if used for assessing BMD before implant placement, it may assist in selecting the patients and early referral for management of osteoporosis. Studies evaluating the accuracy of CBCT to determine BMD have given conflicting results owing to small sample size, each of these studies measuring different bone regions, some of the studies use just radiomorphometric analysis, and some just voxel values.^6,7^

Hence, the present study was planned to assess the diagnostic accuracy of CBCT-based radiomorphometric analysis and radiographic density (voxel values) at different jawbone sites in identifying patients with low BMD. These CBCT-derived parameters were correlated with calcaneal QUS measurements, along with serum calcium and vitamin D levels, in elderly patients.

The study aimed to evaluate and correlate osteoporosis-related changes in the jawbone using CBCT and calcaneal QUS and to further assess their association with biochemical markers of bone metabolism. Specifically, the objectives were to measure BMD at various jaw sites using CBCT voxel values and radiomorphometric indices: (1) to determine the relationship between CBCT voxel values and QUS scores in osteoporotic and non-osteoporotic individuals; (2) to evaluate the correlation between CBCT radiomorphometric parameters and QUS scores; (3) to establish CBCT voxel value thresholds in different jaw regions that may indicate osteoporosis for the studied CBCT unit and population; and (4) to assess the relationship of serum calcium and vitamin D levels with CBCT- and QUS-derived measurements.

## 2. METHODOLOGY

This cross-sectional comparative study included 86 subjects who underwent CBCT for various oral conditions at the Department of Oral Medicine and Radiology, College of Dental Sciences and Bapuji Dental College and Hospital, Davangere.

Participants were divided into two groups based on calcaneal QUS T-scores: a normal group and an osteoporosis group.

Inclusion criteria for the osteoporosis group comprised T-scores ≤ –2.5, and for the normal group, T-scores ≥ –1. Exclusion criteria included pregnancy, smoking, alcohol use, recent calcium or vitamin D supplementation, systemic diseases affecting bone metabolism, and local factors interfering with bone evaluation.

Demographic data were recorded using a structured proforma. This clinical study was conducted according to the principles of the Helsinki Declaration (9th version, 2013). Ethical approval was obtained from the institutional ethics committee, and informed consent was obtained from all participants.

### 2.1 Calcaneus QUS analysis

**Figure 1.**
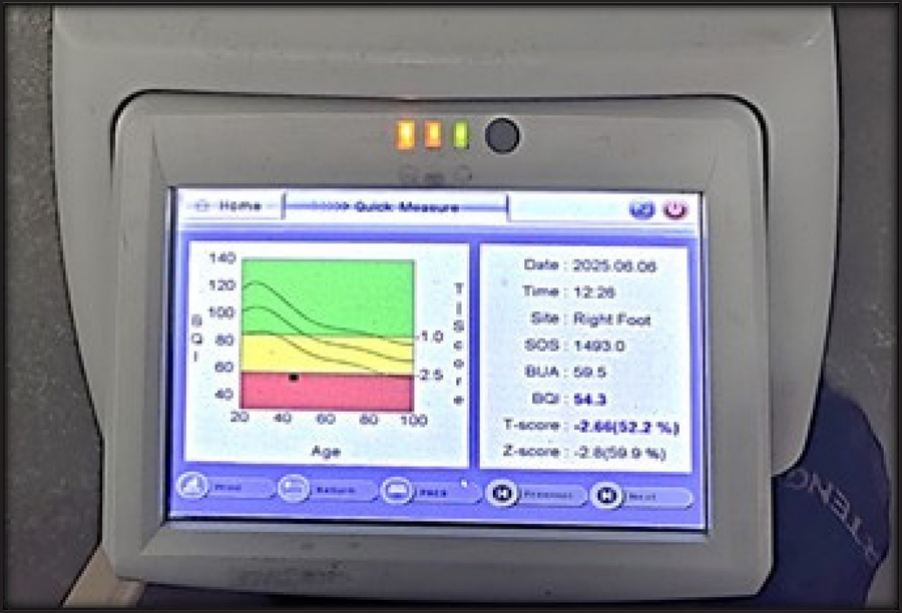
Acquisition of T-score using the calcaneus quantitative ultrasound machine.

The SONOST 3000 (OsteoSys) was used for QUS to assess bone status in the heel. According to the World Health Organization criteria, individuals with QUS measurements (reference test) of calcaneus that yielded T-scores of less than −2.5 were diagnosed with osteoporosis, −1.0 to −2.5 with osteopenia, and participants with T-scores of −1 or greater were considered to have normal bone density (Figure 1).

### 2.2 Serum calcium and vitamin D assessment

After overnight fasting, blood was drawn the next morning for the assessment of serum 25-hydroxy vitamin D and calcium levels.

### 2.3 CBCT analysis

**Figure 2.**
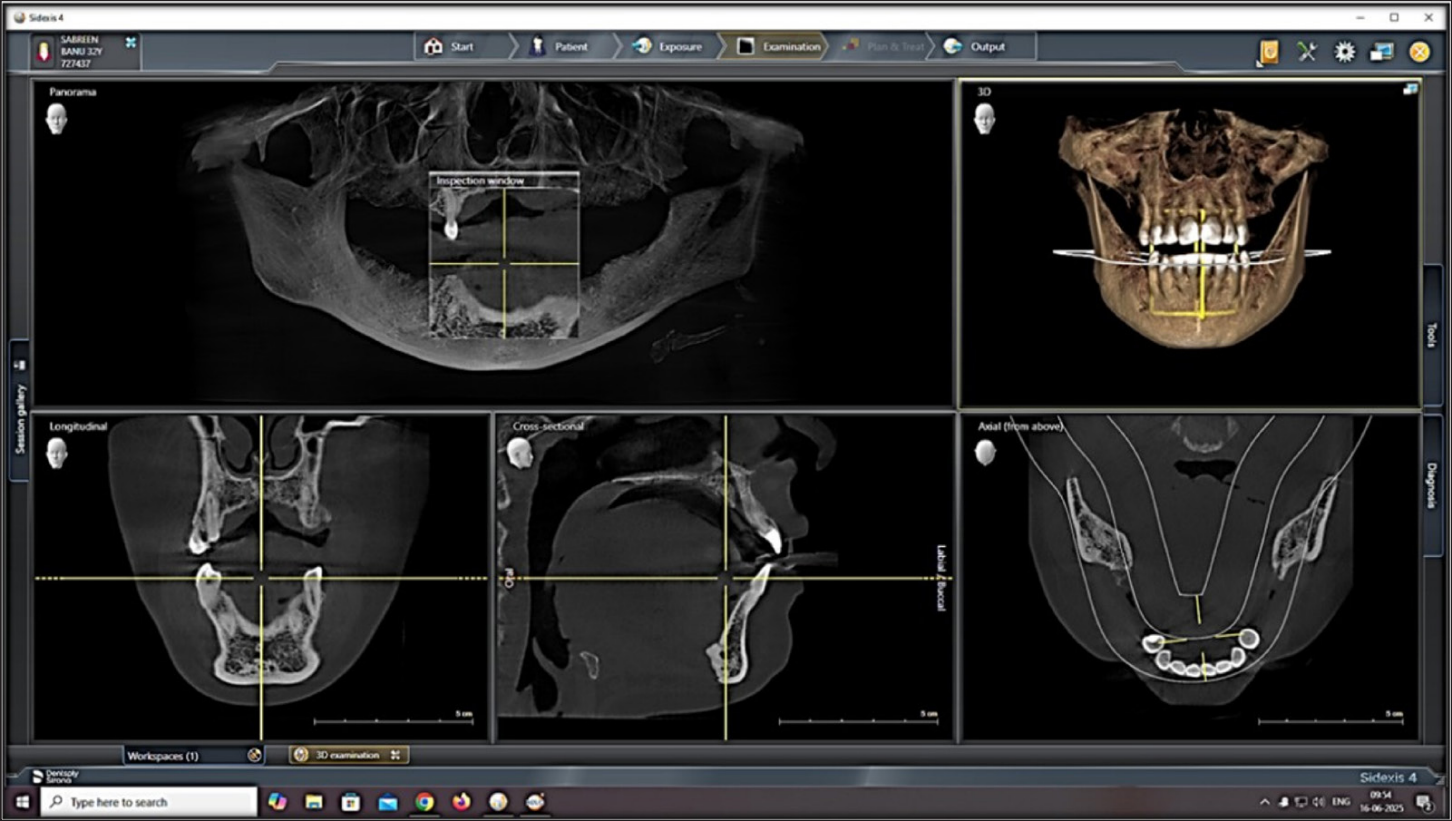
Sidexis image acquisition software.

**Figure 3.**
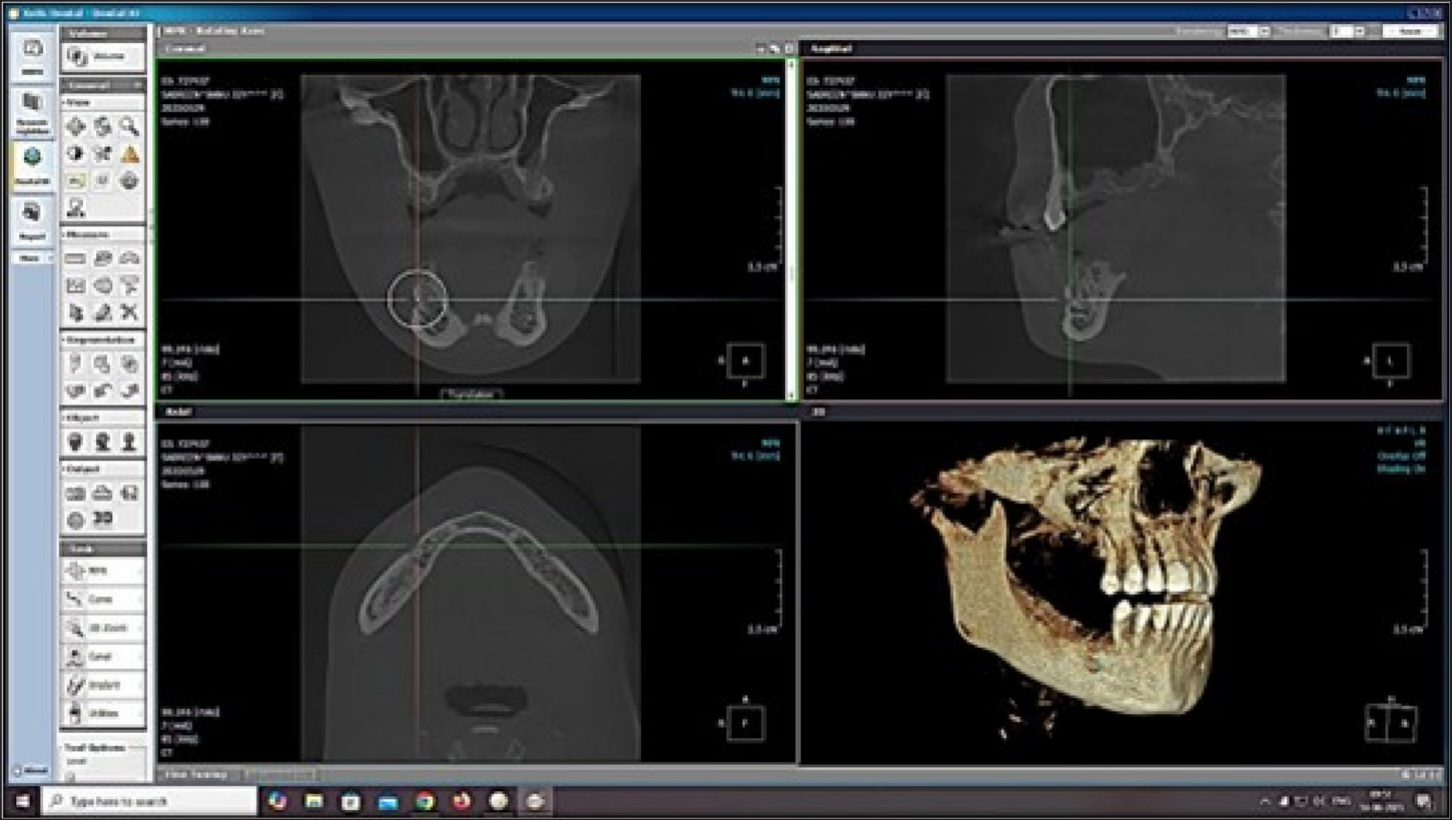
Xelis image analysis software.

CBCT images (index test) were obtained with Orthophos SL 3D by Dentsply Sirona, Germany, using Sidexis 4 X-ray software (Figure 2). Images were analyzed with the Xelis 3D software (Infinitt Healthcare), using <0.1 mm slice thickness (Figure 3). CBCT images of jaw bones were evaluated using radiomorphometric indexes and gray scale (GS) values.

### 2.4 CBCT radiomorphometric measurements

The terms “CTI(S),” “CTI(I),” and “CTMI” on CBCT images were obtained, as defined by Koh et al.^8^

#### 2.4.1 CTI(S)

**Figure 4.**
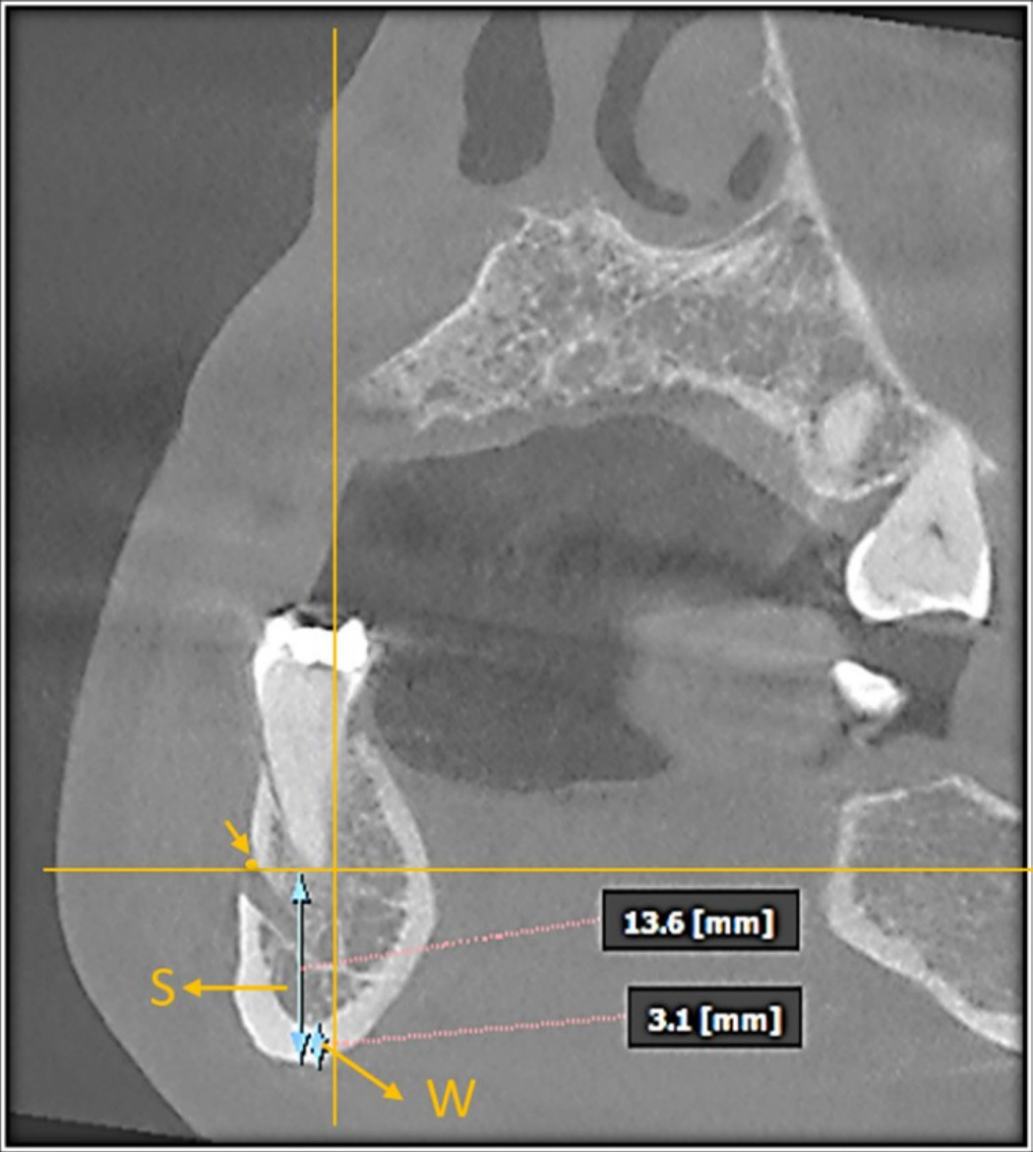
CTI(S) - computed tomography mandibular index (superior).

Computed tomography mandibular index (superior; CTI(S)) is the ratio of the inferior cortical width to the distance from the superior margin of the mental foramen to the inferior border of the mandible (Figure 4).


CTI(S)=W/S

#### 2.4.2 CTI(I)

**Figure 5.**
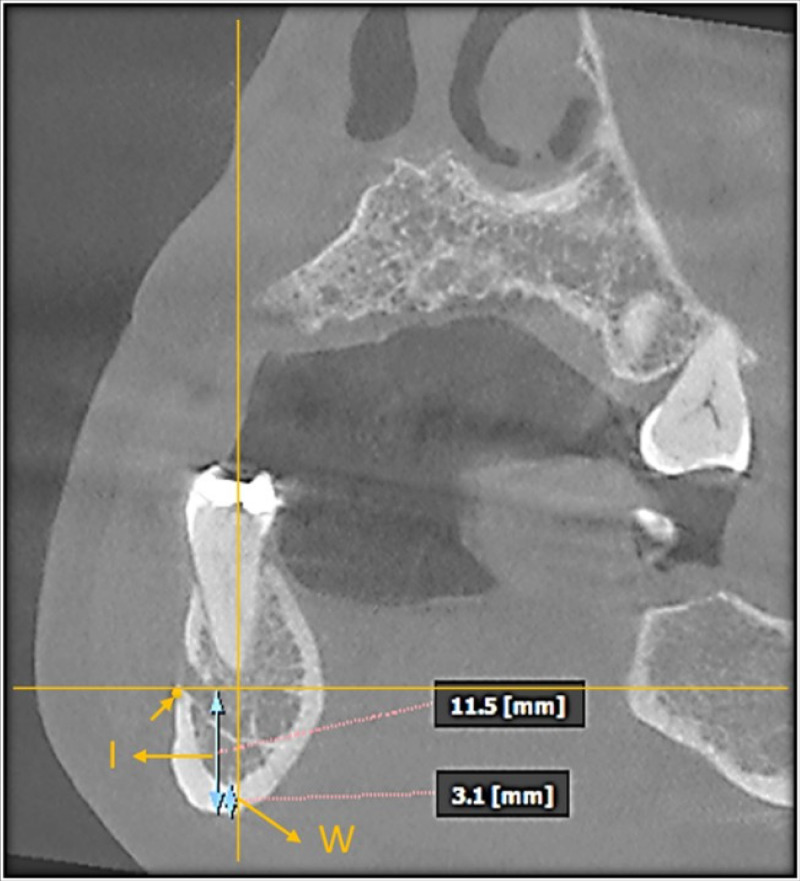
CTI(I) - computed tomography mandibular index.

Computed tomography mandibular index (inferior; CTI(I)) is the ratio of the inferior cortical width to the distance from the inferior margin of the mental foramen to the inferior border of the mandible (Figure 5).


CTI(I)=W/I

#### 2.4.3 CTMI

**Figure 6.**
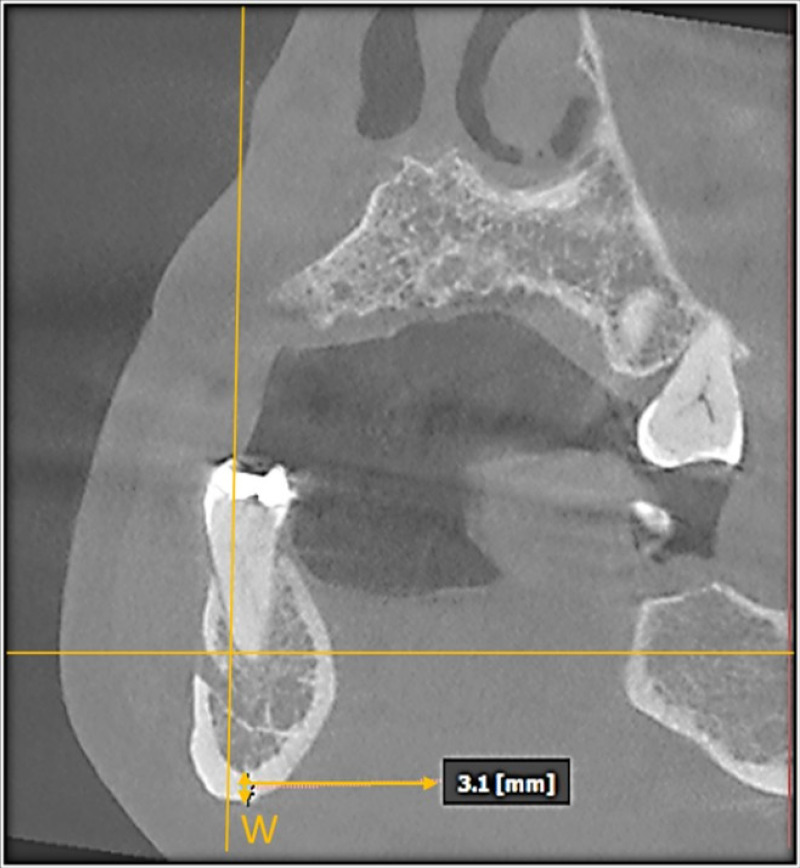
CTMI - computed tomography mental index.

Computed tomography mental index (CTMI) is the inferior cortical width of the mandible (Figure 6).


CTMI=W

#### 2.4.4 CTCI

**Figure 7.**
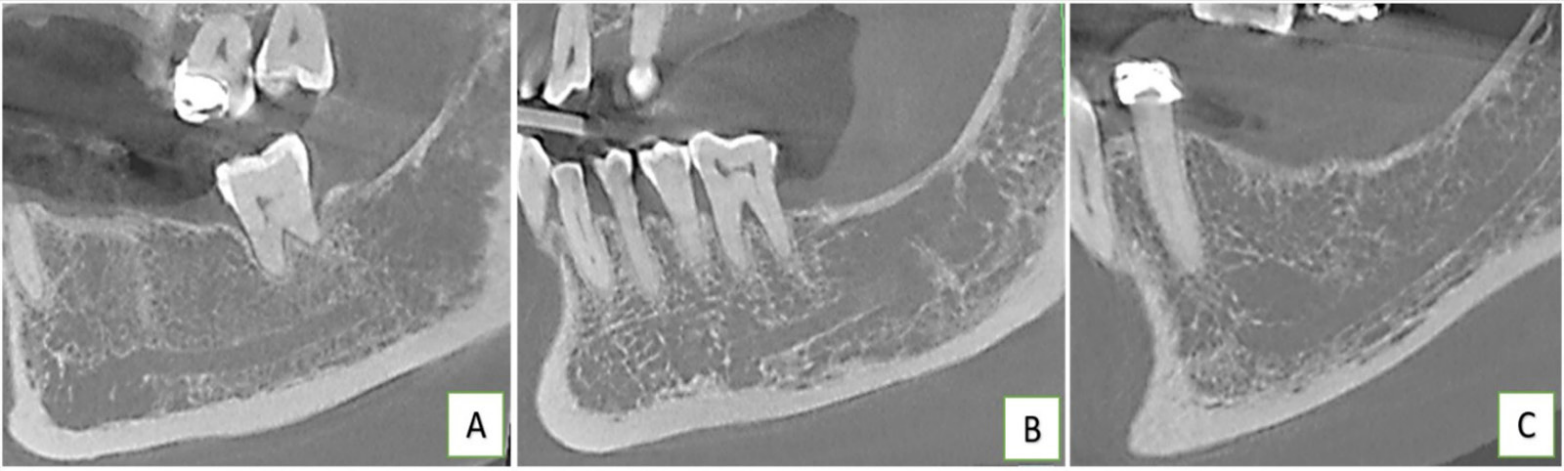
CTCI - computed tomography cortical index types. (A) Type 1 CTCI (B) Type 2 CTCI (C) Type 3 CTCI.

Computed tomography cortical index (CTCI) represents the type of mandibular inferior cortex of the mandible. It was evaluated from the non-orthogonal sagittal image obtained after slice preparation. The morphology of the mandibular inferior cortex was visually examined distal to the mental foramen bilaterally and classified using Klemetti’s^9^ classification as follows (Figure 7):

- CTCI type 1: The endosteal margin of the inferior cortex was smooth on both ends.
- CTCI type 2: The endosteal margin showed semilunar defects or appeared to form endosteal cortical residues.
- CTCI type 3: The cortex was obviously porous with dense endosteal residues.

**Figure 8.**
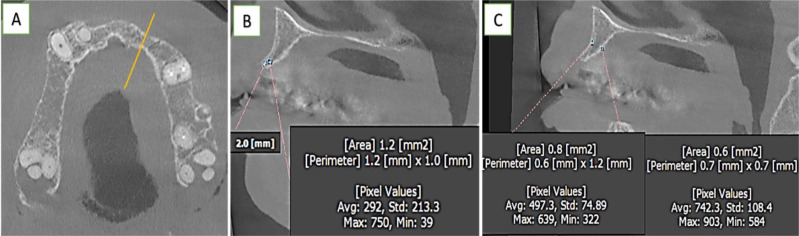
Anterior maxillary voxel values. (A) Yellow line on the axial view indicates the location of the cross-sections. (B) Voxel value 2mm from alveolar crest. (C) Voxel values at labial and palatal cortical bone.

**Figure 9.**
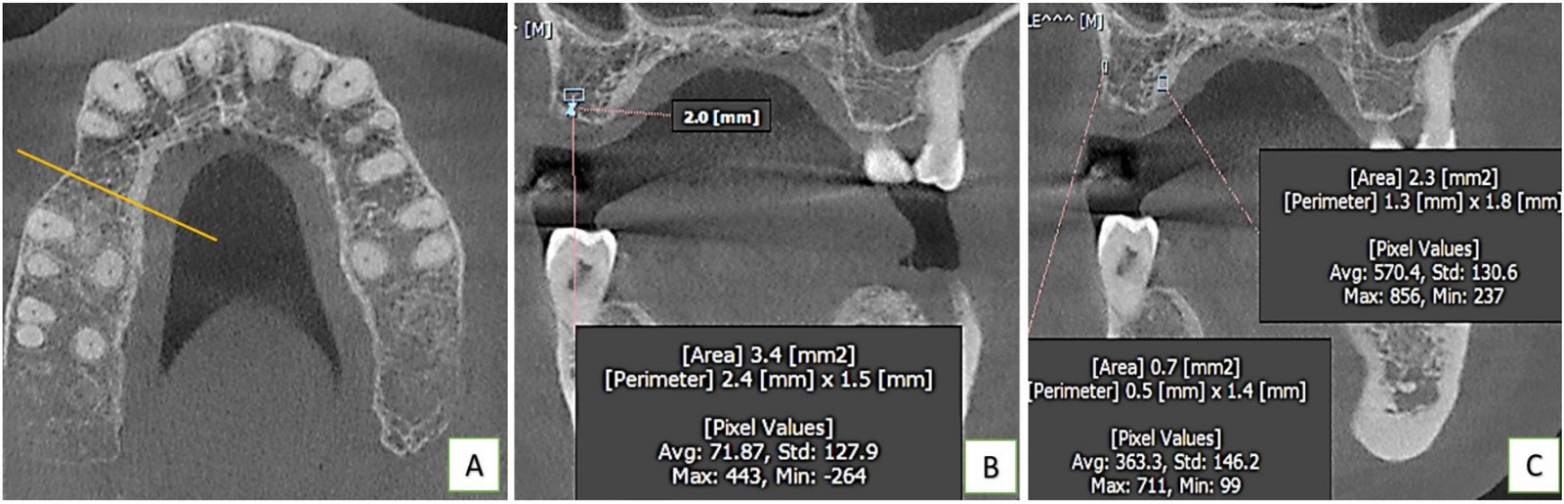
Posterior maxillary voxel values. (A) Yellow line on the axial view indicates the location of the cross-sections. (B) Voxel value 2mm from alveolar crest. (C) Voxel values at buccal and palatal cortical bone.

**Figure 10.**
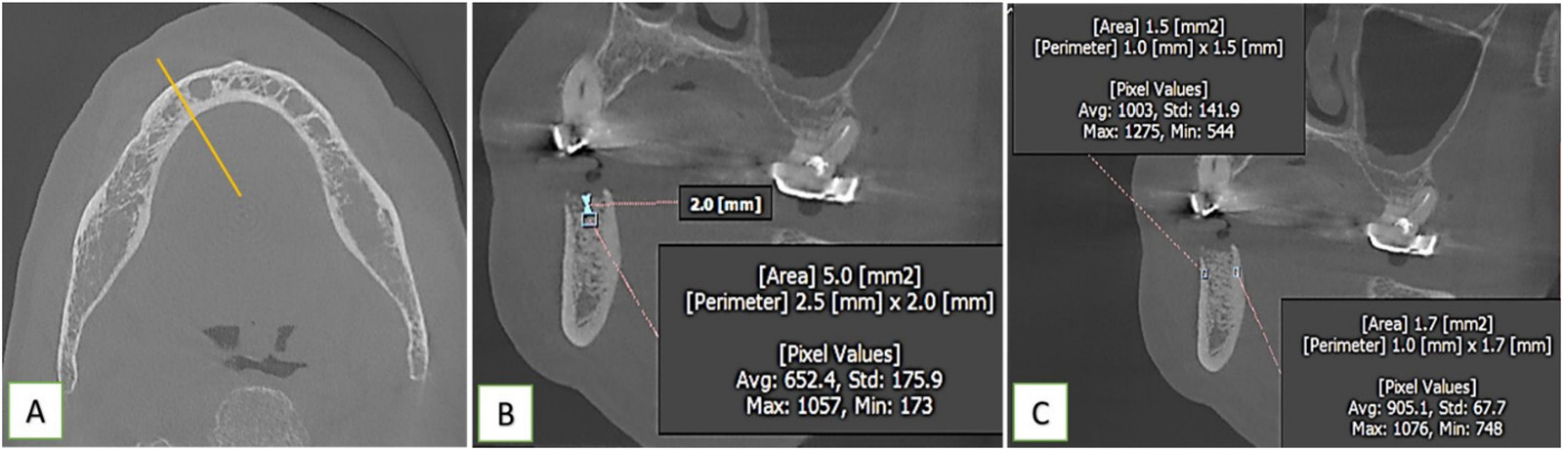
Anterior mandibular voxel values. (A) Yellow line on the axial view indicates the location of the cross-sections. (B) Voxel value 2mm from alveolar crest. (C) Voxel values at labial and lingual cortical bone.

**Figure 11.**
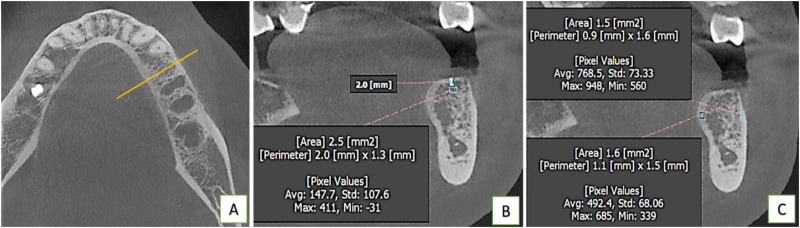
Posterior mandibular voxel values. (A) Yellow line on the axial view indicates the location of the cross-sections. (B) Voxel value 2mm from alveolar crest. (C) Voxel values at buccal and lingual cortical bone.

CBCT voxel or GS values:

For each participant, the GS values were calculated at four regions: namely, anterior maxilla (Figure 8), posterior maxilla (Figure 9), anterior mandible (Figure 10), and posterior mandible (Figure 11).

**Figure 12.**
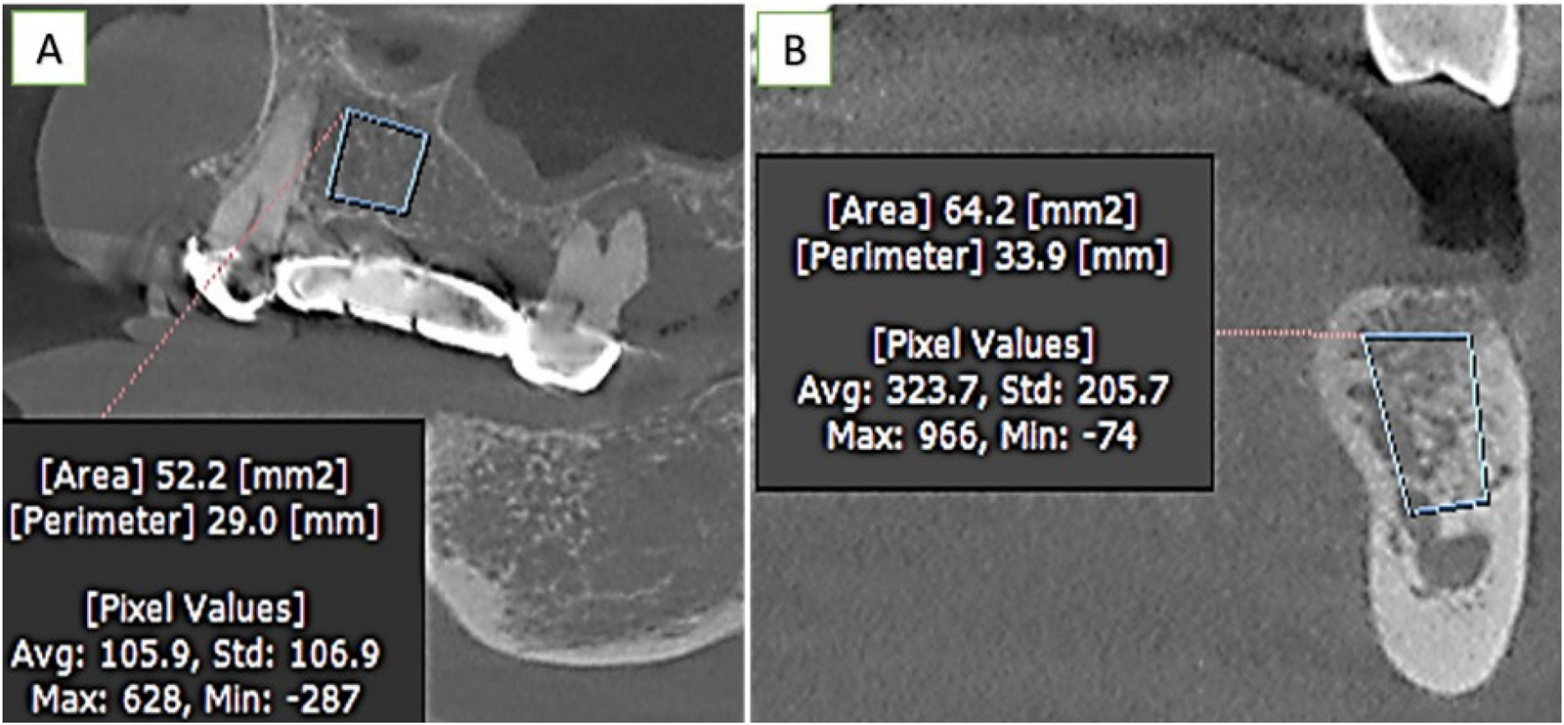
Maxillary and mandibular cancellous bone voxel values. (A) Maxillary cancellous bone voxel values. (B) Mandibular cancellous bone voxel values.

The maxillary voxel values were obtained by selecting two single fields anteriorly and posteriorly on the right and left sides that did not include the cancellous bone in the area. Evaluation of the mandibular cortical area was performed by first selecting a single field on either side that did not extend into the cancellous area, where the right and left side mental foramen were clearly visible in the cortical area on CBCT images. Voxel values were obtained for just cancellous bone by selecting fields on the right and left sides of both maxilla and mandible (Figure 12).

The T-score from the reference test was not available to the performers of the index test but was provided directly to a statistician, as submitted by each participant who underwent the reference test. The assessors of the reference test were not aware of the clinical presentation and voxel values of the participants. All measurements were made bilaterally for each patient, and the mean values were used for further statistical analysis.

### 2.5 Statistical analysis

Data were entered into Microsoft Excel and analyzed using IBM SPSS Statistics v23. Continuous variables were reported as mean ± standard deviation. Independent samples t-tests compared CBCT voxel values, serum calcium, and vitamin D between groups. Pearson’s correlation coefficients assessed relationships between QUS T-scores and CBCT indices, as well as biochemical markers. A *P*-value < 0.05 was considered statistically significant.

## 3. RESULTS

**Figure 13.**
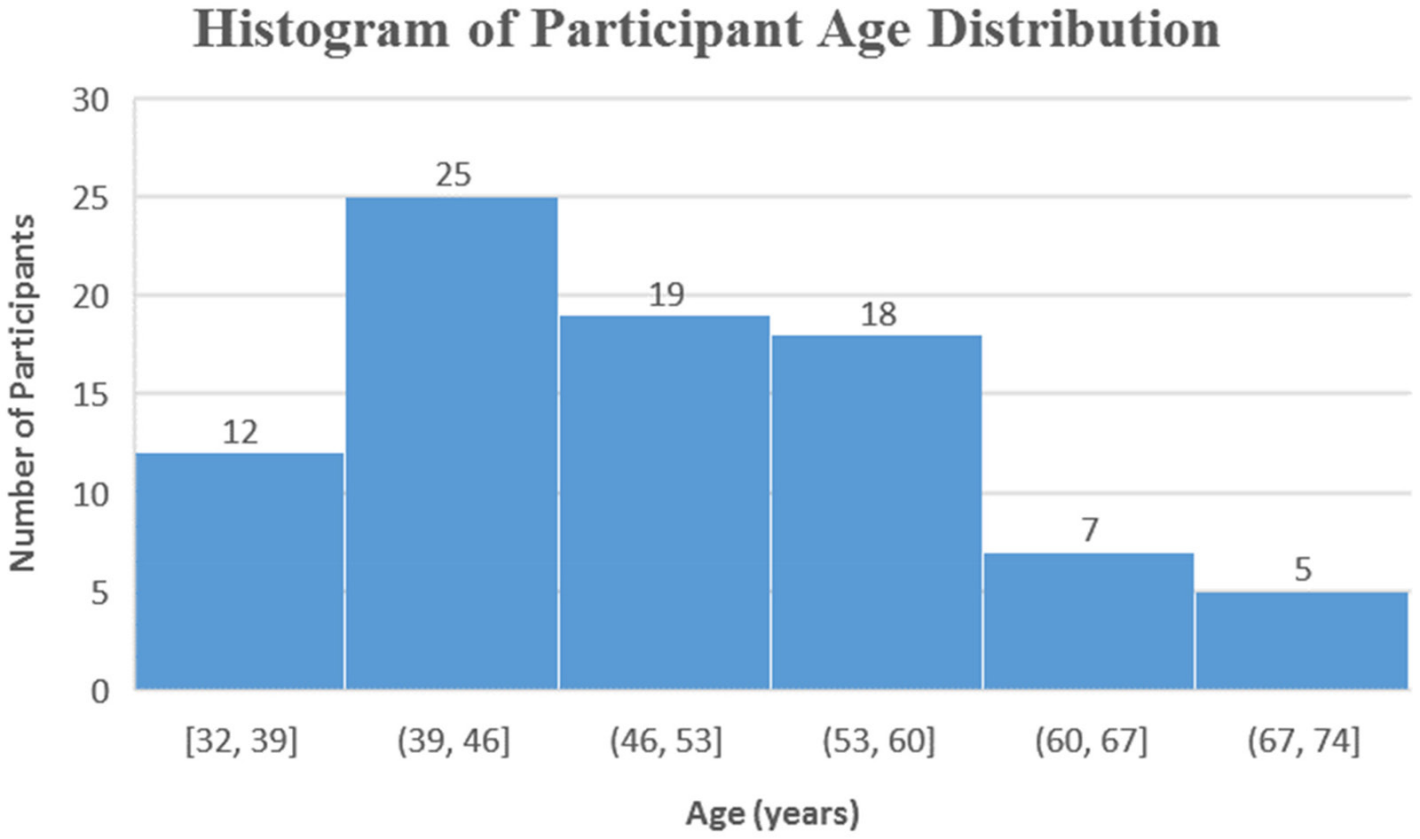
Overall distribution of participant age.

**Figure 14.**
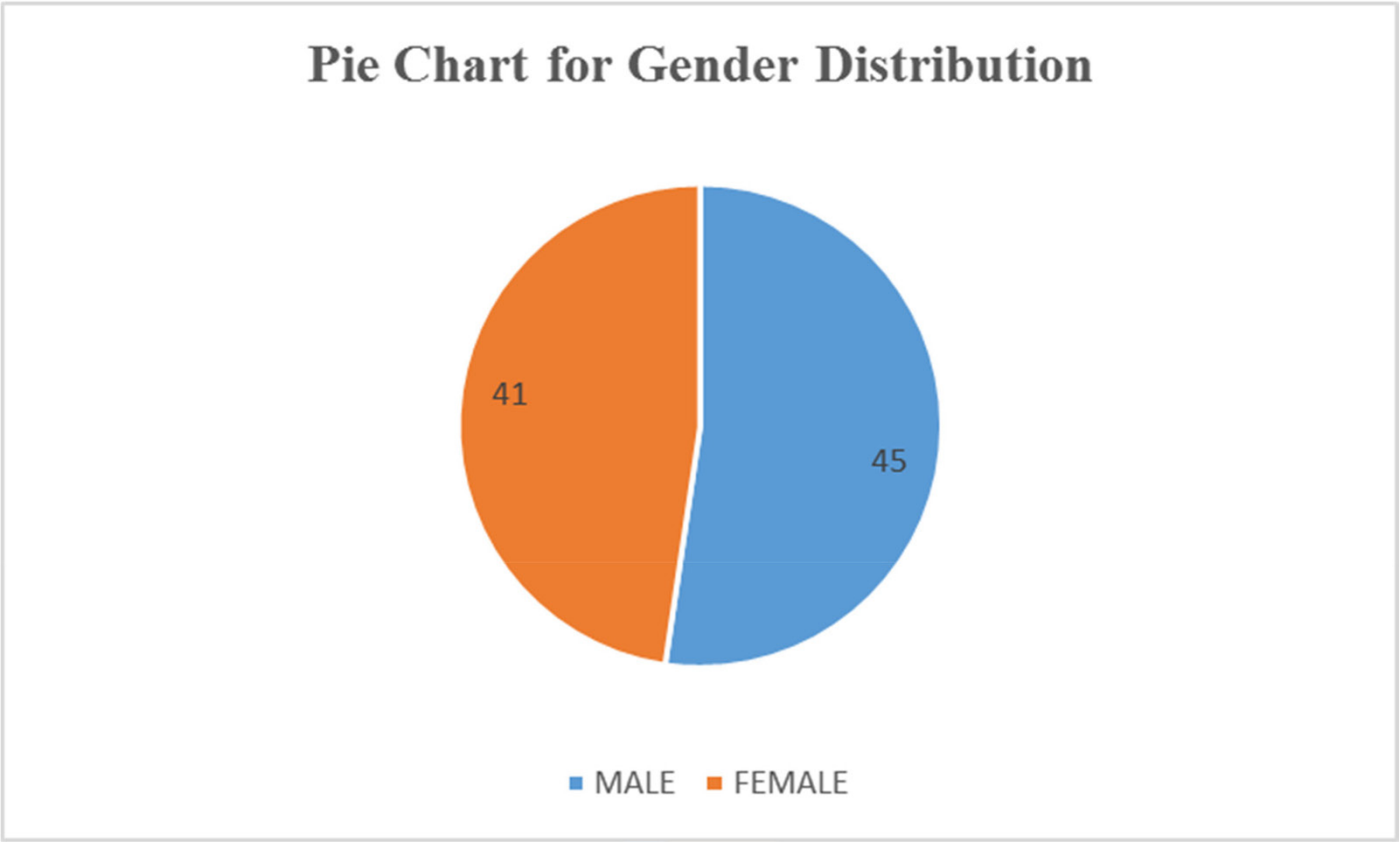
Distribution of participant gender.

**Figure 15.**
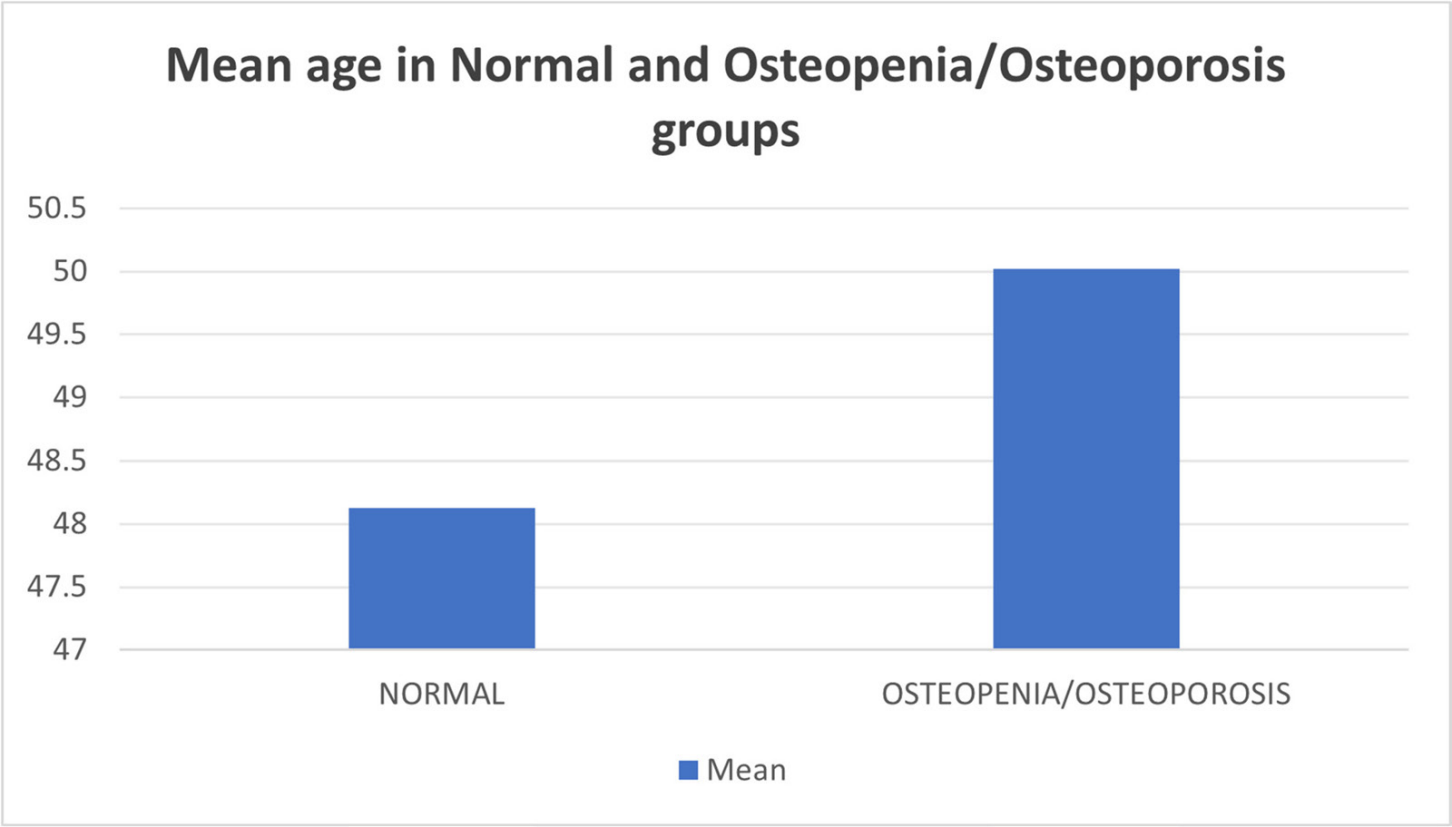
Bar chart showing mean age in normal and osteopenia/osteoporosis groups with error bars representing 95% confidence intervals.

**Figure 16.**
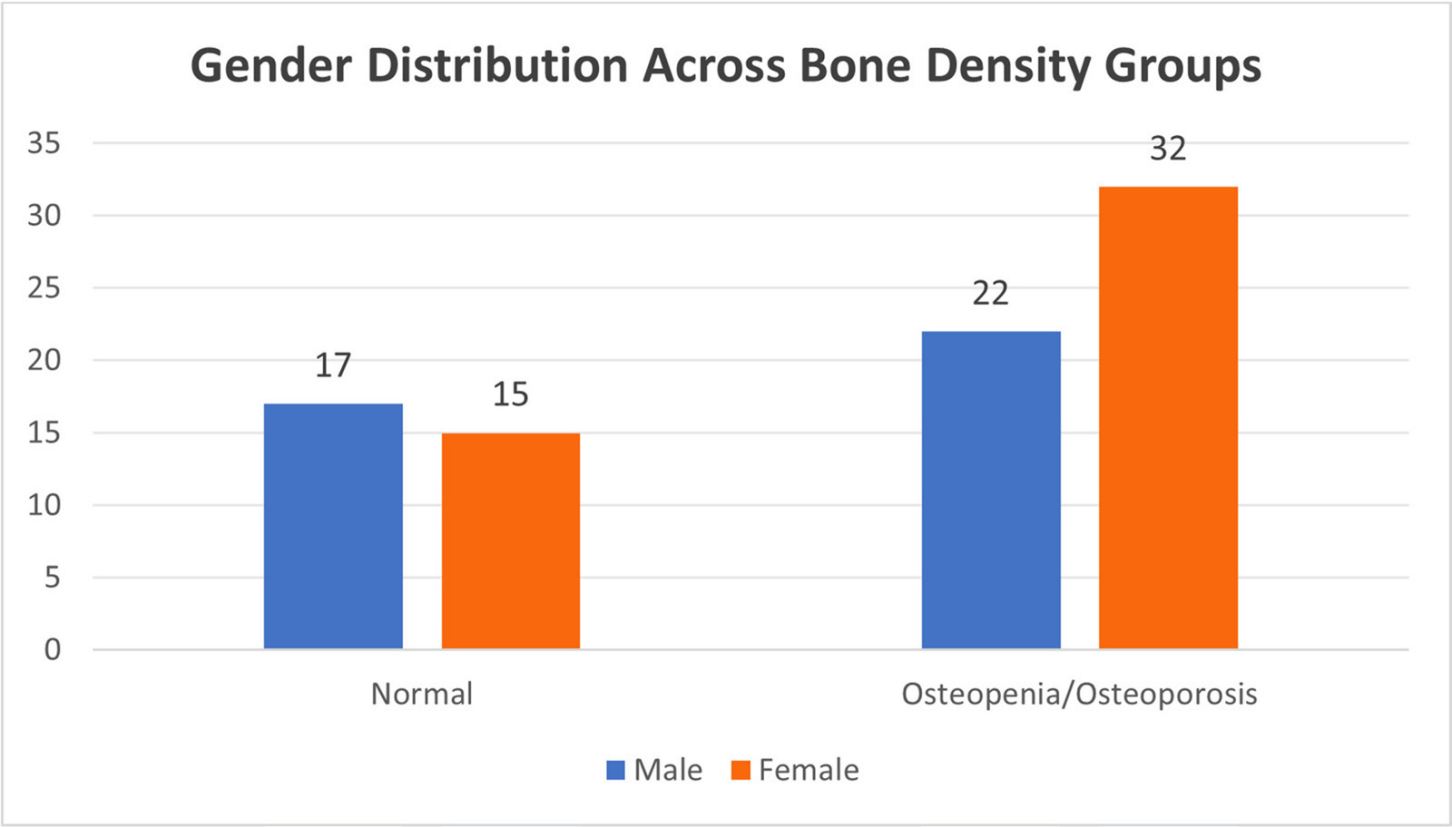
Clustered bar chart showing distribution of male and female participants across normal and osteopenia/osteoporosis groups.

A total of 86 participants (out of 100 initially recruited) were included in the final analysis, as some did not report for the QUS assessment. The mean age was 49.72 ± 9.48 years (range, 32–69 years), with a slight female predominance (47 females, 39 males; Figure 13). There were no statistically significant differences in age (*P* = 0.322) or gender distribution (*P* = 0.265) between the normal and osteopenia/osteoporosis groups (Figures 14–16).

**Figure 17.**
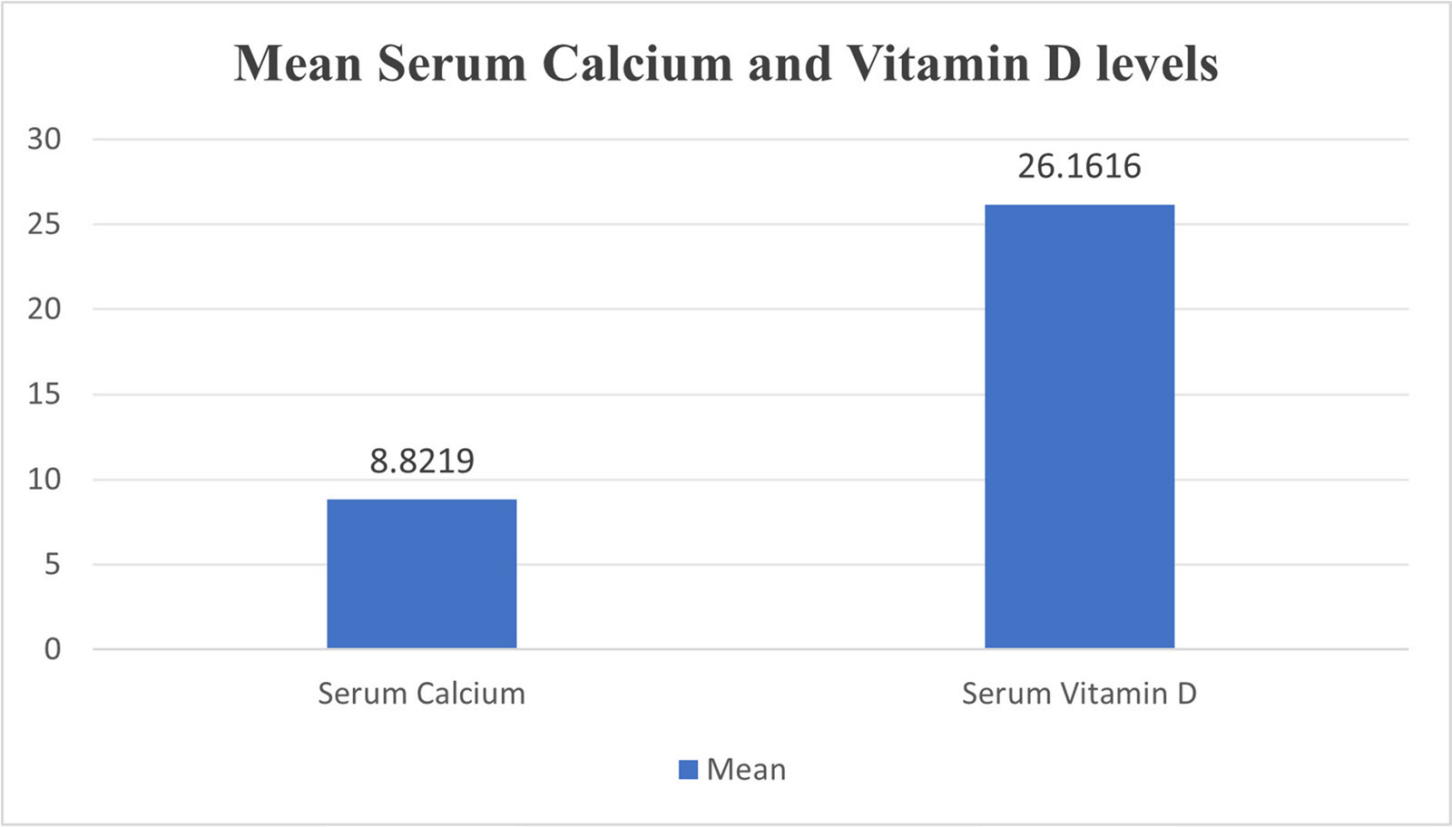
Mean serum calcium and vitamin D levels.

The mean QUS T-score was −1.12 ± 1.15, indicating a substantial proportion of participants with reduced BMD. Mean serum calcium and vitamin D levels were 8.82 ± 1.13 mg/dL and 26.16 ± 9.43 ng/mL, respectively (Figure 17). Mean CBCT-derived voxel values were 756.28 ± 115.59 for cortical bone and 318.03 ± 118.58 for cancellous bone.

### 3.1 Comparison between QUS-defined groups

**Figure 18.**
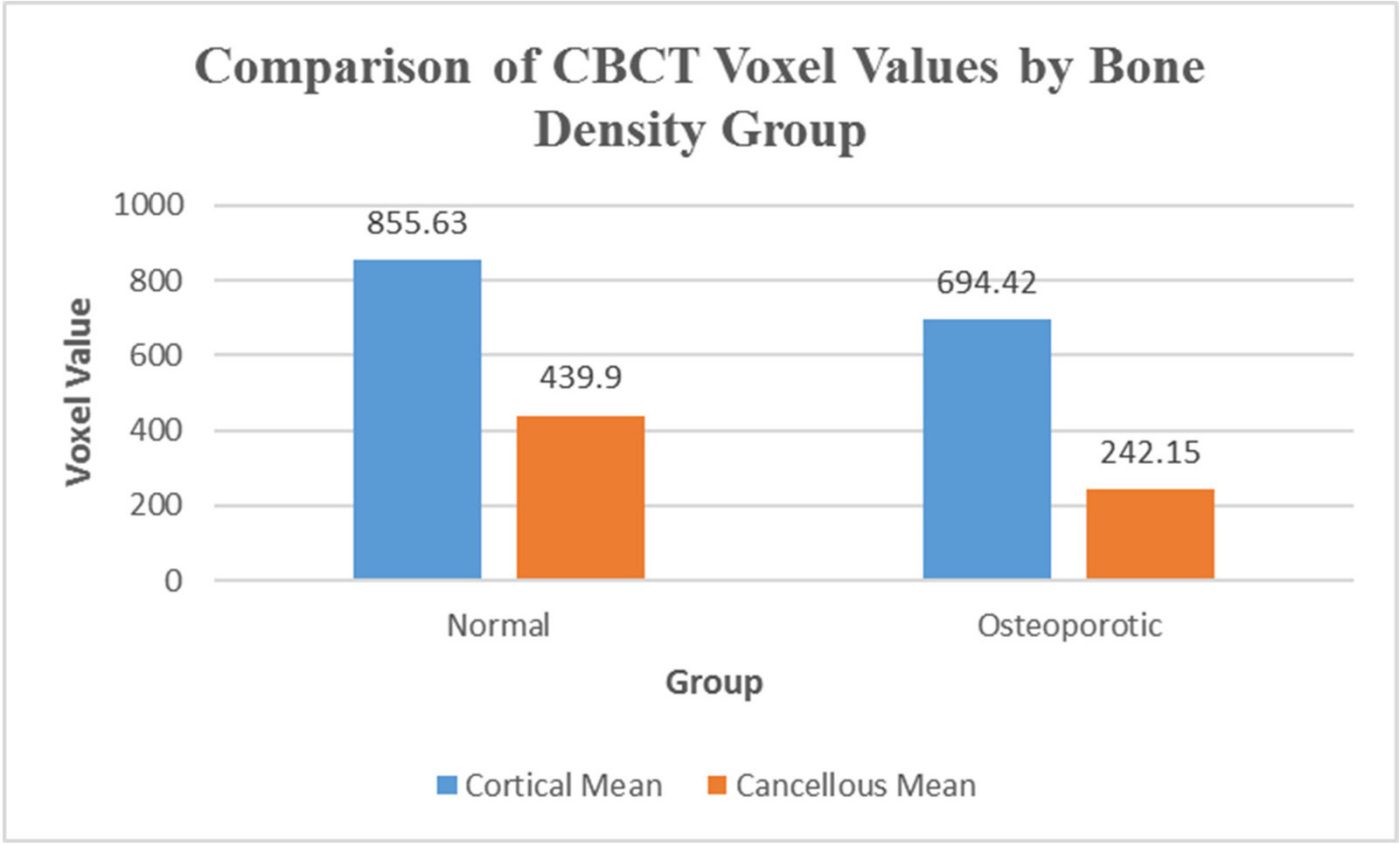
Comparison of average cortical and cancellous CBCT voxel values between normal and osteoporosis groups (based on QUS T-scores).

**Figure 19.**
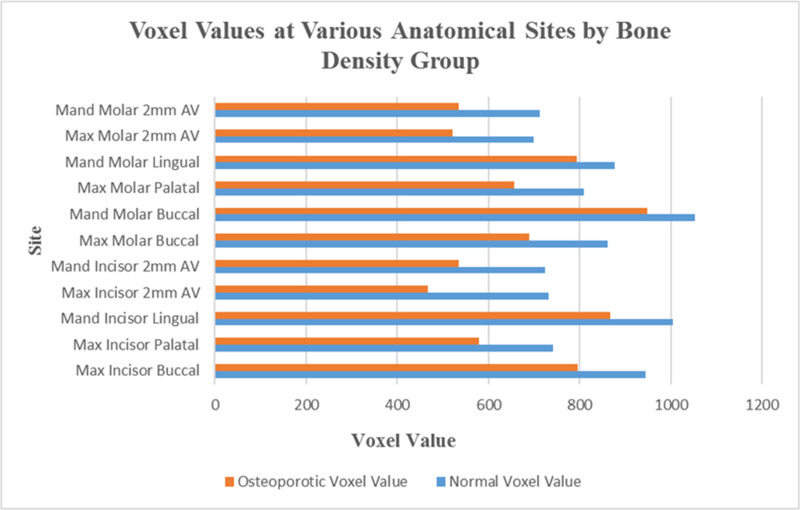
Voxel values by anatomical site and bone density group.

Participants with normal bone density demonstrated significantly higher CBCT-derived voxel values compared to the osteopenia/osteoporosis group. Mean cortical voxel values were 855.63 ± 71.38 in the normal group versus 694.42 ± 92.42 in the osteoporosis group, while cancellous voxel values were 439.90 ± 85.61 and 242.15 ± 56.43, respectively (*P* < 0.001; Figure 18). Region-specific analysis showed consistently higher cortical and cancellous voxel values across both anterior and posterior maxillary and mandibular sites in the normal group (Figure 19).

Serum calcium levels were higher in the normal group but did not reach statistical significance (*P* = 0.088). In contrast, serum vitamin D levels were significantly higher in the normal group (36.55 ± 5.35 ng/mL) compared to the osteopenia/osteoporosis group (20.00 ± 6.80 ng/mL; *P* < 0.001).

### 3.2 Correlation analysis

**Figure 20.**
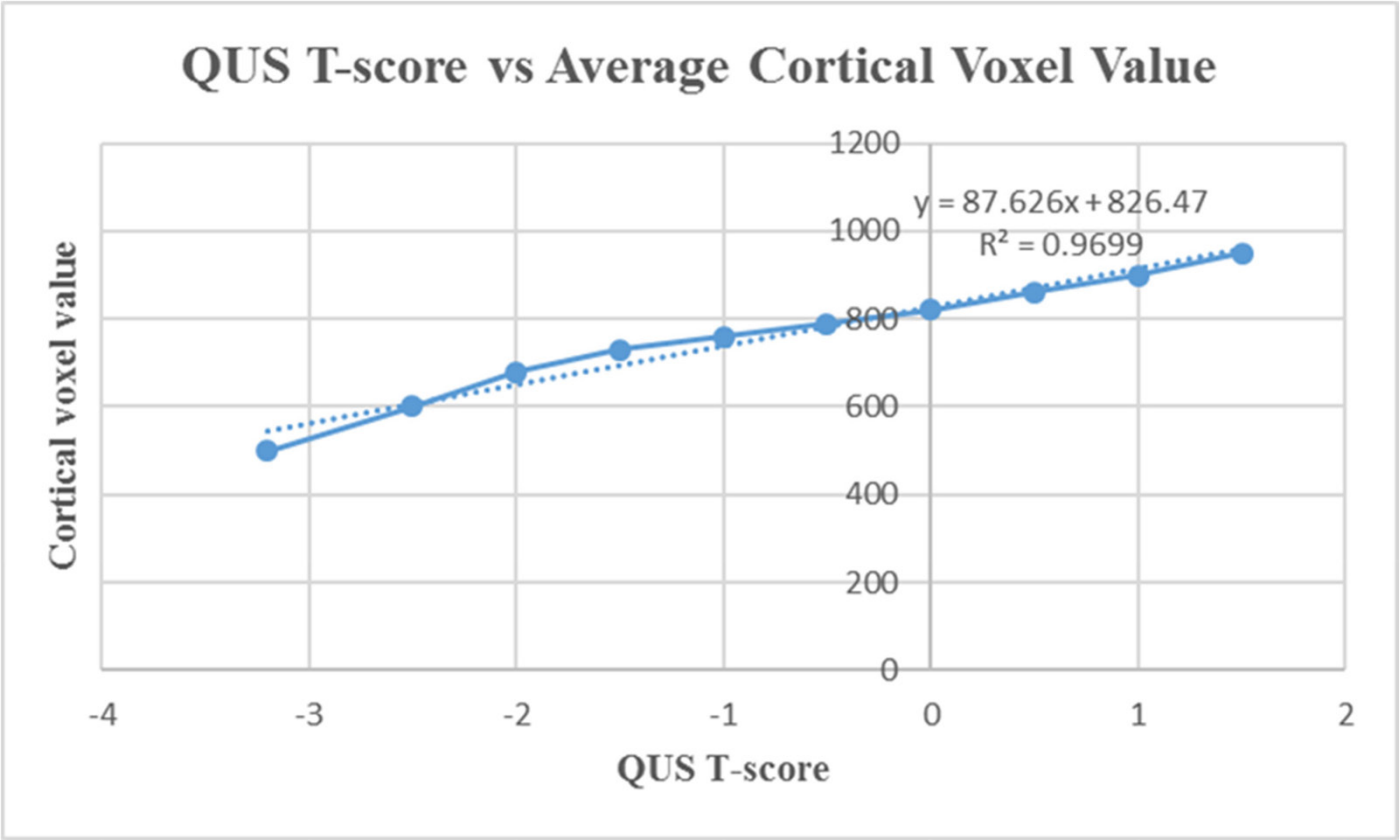
Correlation between average cortical voxel value and QUS T-score.

Pearson correlation analysis revealed strong, statistically significant positive correlations between QUS T-scores and CBCT-derived voxel values for both cortical (*r* = 0.766, *P* < 0.001) and cancellous bone (*r* = 0.789, *P* < 0.001; Figure 20). Serum vitamin D levels also showed a strong positive correlation with QUS T-scores (*r* = 0.695, *P* < 0.001), while serum calcium demonstrated a weaker but significant correlation (*r* = 0.243, *P* = 0.024).

Site-specific analysis demonstrated significant positive correlations between QUS T-scores and cortical voxel values at multiple maxillary and mandibular anterior and posterior regions (*P* < 0.01). In contrast, CBCT-derived morphometric indices, including CTMI, CTI (W/S), and CTI (W/I), did not show significant correlations with QUS T-scores (*P* > 0.05).

### 3.3 Cortical index analysis

Spearman’s rank correlation demonstrated a strong negative association between CTCI type and QUS T-score (*P* = −0.682, *P* < 0.001), indicating that as the erosion grade increases (e.g., to Type 3), the bone density (T-score) decreases.

## 4. DISCUSSION

Osteoporosis represents a severe public health burden in India, characterized by a significantly earlier onset compared to Western populations. In India, the burden is particularly high, with an estimated 35% to 50% of women and 20% of older men affected by the disease.^8,10^ Additionally, Indian women typically experience menopause between the ages of 46 and 48 years, nearly a decade earlier than their Western counterparts,^10,11^ resulting in prolonged estrogen deficiency that accelerates bone loss.^7^ This early onset is further exacerbated by widespread nutritional deficiencies, particularly in calcium and vitamin D.^12,13^ While DEXA remains the gold standard for diagnosis, its cost and limited accessibility^14,15^ have led to the increased utility of QUS of the calcaneus. An alternative to a gold standard, often referred to as an imperfect reference standard or best available reference, has been used to compare the diagnostic accuracy because an error-free “gold standard that is DEXA” was unavailable, too expensive, and impractical to implement in our study.

Yen et al. conducted a pre-screening analysis and reported that combining QUS with clinical risk factors improved the identification of patients requiring further DEXA evaluation.^16^ Although not a substitute, QUS has emerged as a validated, radiation-free alternative for population screening,^17,18^ providing critical insights into bone quality and strength that correlate well with the mechanical properties of trabecular bone.

The present study aimed to assess the diagnostic accuracy of CBCT by correlating radiomorphometric analysis and voxel values with QUS T-scores and biochemical markers. Our findings revealed a strong, statistically significant positive correlation between QUS T-scores and CBCT-derived voxel values for both cortical and cancellous bone. Participants in the normal bone density group exhibited significantly higher voxel values in both anterior and posterior jaw regions compared to those in the osteopenia/osteoporosis group. Although CBCT voxel values (GS values) lack the standardized calibration of Hounsfield Units used in conventional CT due to technical differences in X-ray beam quality, scatter, detector sensitivity, and reconstruction algorithms of a specific machine used,^19^ these results support previous research such as studies by Naitoh et al.^20^ and Nomura et al.^21^ suggesting that GS values can serve as effective semi-quantitative estimators of BMD when correlated with systemic bone health. Although the absence of a calibration phantom limits standardization, it is acceptable in correlation-based studies performed using a single CBCT system.

Regarding biochemical markers, the study highlighted a significant divergence between serum vitamin D and calcium levels in relation to bone density. Serum vitamin D levels demonstrated a strong positive correlation with QUS T-scores, with the osteoporosis group exhibiting significantly lower levels than the normal group. Conversely, serum calcium levels showed a weaker association and no statistically significant difference between the study groups. Krieg et al.^22^ in a longitudinal study in elderly institutionalized women demonstrated that supplementing vitamin D3 (440 IU × 2/day) and calcium (1000 mg/day) led to a statistically significant increase in calcaneal QUS broadband ultrasound attenuation (BUA) after 2 years, while the control group experienced a decline. This maintenance of normal serum calcium levels in osteoporotic patients is likely attributable to homeostatic mechanisms; specifically, insufficient dietary calcium triggers increased parathyroid hormone (PTH) secretion. This process mobilizes calcium from the skeletal system to maintain systemic levels, thereby masking the deficiency in blood tests while aggressively resorbing bone.^23^

The assessment of radiomorphometric indices yielded mixed diagnostic utility. The CTCI, which qualitatively categorizes mandibular cortical erosion based on Klemetti’s^9^ criteria, showed a strong negative correlation with QUS T-scores, as the erosion. This confirms that increased cortical porosity (Type 3) is a reliable indicator of compromised systemic BMD. However, quantitative indices such as CTMI and CTI ratios did not demonstrate statistically significant correlations with QUS T-scores. This suggests that while qualitative assessment of cortical integrity is valuable, specific quantitative morphometric measurements may be unreliable markers of systemic bone health due to susceptibility to variations in machine settings, artifacts, and anatomical differences.

Several limitations should be acknowledged, including that CBCT voxel values are machine-dependent and not standardized, QUS was used instead of DEXA (gold standard), cross-sectional design limits causal inference, absence of calibration phantom affects reproducibility, and the sample size was modest.

Despite these limitations, this study validates the potential of CBCT-derived voxel values and the qualitative CTCI as non-invasive, adjunctive tools for opportunistic osteoporosis screening. When combined with the assessment of serum vitamin D levels, these parameters can assist dental practitioners in identifying high-risk patients who may require referral for comprehensive bone health evaluation, helping to address the significant underdiagnosis of osteoporosis in the Indian population.

## 5. CONCLUSION

A significant association was observed between serum vitamin D levels and both QUS and CBCT indicators of bone density, reinforcing its role in skeletal health. CBCT-derived voxel values showed strong correlations with QUS T-scores, with significantly higher cortical and cancellous values in participants with normal bone density compared to those with osteopenia or osteoporosis. Similar region-specific trends were noted in both anterior and posterior jaw regions, supporting the utility of CBCT in identifying compromised bone density.

In contrast, CBCT morphometric indices, including cortical thickness and CTI ratios, did not demonstrate significant correlations with QUS scores, suggesting limited diagnostic value when used alone. A strong negative correlation between CTCI type and QUS T-scores indicated that increased mandibular cortical erosion was associated with lower BMD. While serum calcium showed weaker associations with bone density, biochemical analysis confirmed that low vitamin D levels were consistently associated with reduced BMD and lower CBCT voxel values.

Overall, the findings highlight the value of a multimodal diagnostic approach combining CBCT, QUS, and biochemical markers for early identification of individuals at risk for osteopenia or osteoporosis. Future large-scale, longitudinal studies integrating standardized CBCT calibration protocols and validation against DEXA are warranted to enhance the reliability and clinical translation of these findings.

## ACKNOWLEDGEMENTS

The authors would like to thank the staff of the Department of Oral Medicine and Radiology, College of Dental Sciences and Bapuji Dental College and Hospital, Davangere, for their support and assistance during the conduct of this study. The authors also express their sincere gratitude to all the participants involved in this research.

## DISCLOSURE OF AI USE

The authors used AI-assisted tools solely for language refinement and grammatical editing during manuscript preparation. The authors take full responsibility for the content of the manuscript.

## AUTHOR CONTRIBUTIONS

All authors contributed to this work, including conceptualization, methodology, data curation, software and validation, investigation and analysis, visualization, writing – original draft, writing – review and editing, resources, supervision, project administration, and funding acquisition.

## CONFLICT OF INTEREST

The authors declare that they have no conflict of interest.

## FUNDING

The author(s) received no financial support for the research, authorship, and/or publication of this article.

## DATA AVAILABILITY

The datasets generated and/or analysed during the current study are available from the corresponding author on reasonable request.
